# Fiber-based 3D nano-printed holography with individually phase-engineered remote points

**DOI:** 10.1038/s41598-022-25380-2

**Published:** 2022-12-03

**Authors:** Malte Plidschun, Matthias Zeisberger, Jisoo Kim, Torsten Wieduwilt, Markus A. Schmidt

**Affiliations:** 1 Leibniz Institute of Photonic Technology, Albert-Einstein-Str. 9, 07745 Jena, Germany; 2 Abbe Center of Photonics and Faculty of Physics, Friedrich-Schiller-University Jena, Max-Wien-Platz 1, 07743 Jena, Germany; 3 Otto Schott Institute of Materials Research (OSIM), Friedrich-Schiller-University Jena, Fraunhoferstr. 6, 07743 Jena, Germany

**Keywords:** Fibre optics and optical communications, Optoelectronic devices and components

## Abstract

The generation of tailored light fields with spatially controlled intensity and phase distribution is essential in many areas of science and application, while creating such patterns remotely has recently defined a key challenge. Here, we present a fiber-compatible concept for the remote generation of complex multi-foci three-dimensional intensity patterns with adjusted relative phases between individual foci. By extending the well-known Huygens principle, we demonstrate, in simulations and experiments, that our interference-based approach enables controlling of both intensity and phase of individual focal points in an array of spots distributed in all three spatial directions. Holograms were implemented using 3D nano-printing on planar substrates and optical fibers, showing excellent agreement between design and implemented structures. In addition to planar substrates, holograms were also generated on modified single-mode fibers, creating intensity distributions consisting of about 200 individual foci distributed over multiple image planes. The presented scheme yields an innovative pathway for phase-controlled 3D digital holography over remote distances, yielding an enormous potential application in fields such as quantum technology, life sciences, bioanalytics and telecommunications. Overall, all fields requiring precise excitation of higher-order optical resonances, including nanophotonics, fiber optics and waveguide technology, will benefit from the concept.

## Introduction

The desired creation of arbitrary field patterns with complex spatial distribution is required in many areas of science and applications, including dark-field^1,2^, light-sheet^3,4^ and structured illumination microscopy (SIM)^5,6^, 3D-nanoscale position retrieval^7^, excitation of higher-order fiber modes^8,9^, and coupling to multicore fibers within telecommunication^10^. Some of these applications require the generation of multiple individual foci within one or more focal planes, additionally being relevant in applications such as parallelized 3D nano-printing^11^, simultaneous optical trapping and tracking at multiple locations^12^, and parallel collection of light of diffusing emitters in optofluidics^13,14^. Furthermore, the controllable and reproducible generation of such light patterns in a remote way is another key challenge that has the potential to open further areas of applications.

In addition to concepts that rely on resonant structures such as dielectric metasurfaces^15^ or plasmonics^16^, a widely used approach for creating single—and multi-focus light patterns relies on tailored phase masks in the aperture plane using interference to create the desired focal pattern in the image plane^17,18^. Here, approaches such as amplitude or phase masks are widely employed^18^, with phase holograms showing substantially better efficiency than amplitude masks^18^. An important point is the concrete implementation strategy, which has direct impact on the performance of the respective device: For example, Refs.^18,19^ compare different types of phase plates. The 2-level phase mask, which from a technological standpoint employ the simplest type of fabrication strategy, show limited efficiencies of about 40%. Multi-level phase masks, including continuous kinoform profiles, can achieve much higher efficiencies.

In a typical scenario, only the desired intensity distribution is known without any knowledge of the associated phase, preventing the direct engineering of the phase mask. To address this intrinsic issue, numerical iterative calculation methods such as iterative Fourier transform algorithms IFTAs^20–23^ (e.g., Gerchberg-Saxton algorithm^17,24^) are commonly used. These methods are computationally intensive and require well-chosen input conditions, as the calculated phase distribution strongly depends on the input, hence only yielding a reasonable solution for properly chosen inputs. Furthermore, usually there are no unique solutions and the probability of stagnation of the algorithm when approaching local minima is not negligible. It should be noted that in most cases, iterative approaches are not able to optimize holograms with respect to the desired phase distribution, which is problematic in situations where both intensity and phase or polarization need to be controlled, such as within the excitation of higher order fiber modes.

An alternative approach to obtain holograms for multi-focus patterns relies on direct analytical calculation of the appropriate phase distribution via the Huygens-Fresnel principle: Here, it is assumed that the desired multi-focus pattern in a focal plane is composed of individual diffraction limited spots that are formed via the superposition of spherical waves originating from the aperture plane^25,26^. For the generation of multi-focus distributions, this approach offers important advantages, such as a simple, robust, fast, and reliable calculation of the phase distribution, as well as the existence of only one unique solution. In addition to existing concepts^25,26^, our approach allows the individual phase of each focal point to be predefined, which makes it possible to study the interaction of individual foci. It is important to note here that this particular type of investigation cannot be performed via the usual^17,20–24^ iterative algorithms, as these do not allow the phases to be explicitly defined.

The application of computer-generated holograms (CGHs) over remote distances via using optical fibers is an open and challenging field of research, as the usual implementation methods of CGHs are incompatible with optical fibers. Recent approaches for structuring fiber end-faces include modified electron beam lithography^27,28^, self-assembly^29^, focused ion beam structuring^30^, and fixing nanostructures via gluing^31^. Within this context, 3D nano-printing is an outstanding method being particularly suitable for structuring fiber end-surfaces. Due to the flexibility of this method, a variety of functionalities can be incorporated into fibers, examples of which include sophisticated refractive^32^ and diffractive^33^ lenses and micro-optical components^34,35^, with first attempts for the realization of advanced CGHs that solely modulate the intensity being recently demonstrated^36^. Overall, the combination of fibers and nano-printed holograms represents a very promising approach, especially from the application side.

**Figure 1:**
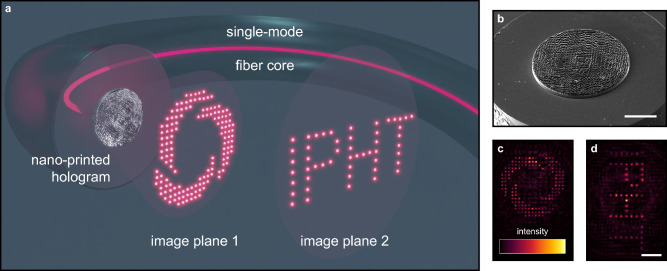
Multi-focus holography on a modified single-mode fiber using a nano-printed phase hologram. (**a**) Scheme of a 3D nano-printed hologram on the end-face of the core of a modified single-mode fiber, together with two multi-focus distributions in two image planes. (**b**) Scanning-electron-micrograph of an example of a nano-printed hologram on the fiber facet (scale bar 20 µm). (**c**) Measured intensity distribution in the first and (**d**) the second focal plane, showing the logo of the Leibniz Institute of Photonic Technology (IPHT) and its lettering (same scale bar (10 µm) for both).

In this work, we address the aspects by presenting a novel route towards generating complex multi-focal three-dimensional intensity patterns with additionally included adjustable relative phases between individual foci as an extension of the well-known Huygens-Fresnel principle^25,26^ both on planar substrates as well as on optical fibers. Using 3D nano-printing and an analytic interference-based design principle, advanced digital holograms that create unconventional multi-spot patterns beyond what can be achieved via iterative algorithms are realized and experimentally demonstrated. In particular, the scenario of opposite phases of adjacent foci in a dual-focus and a ring configuration is intensively studied, where, for example, the separation of two foci below the resolution limit could be shown. Furthermore, intensity distributions consisting of about 200 individual foci distributed over multiple image planes are created on a modified single-mode fiber (Fig. 1).

## Design principle

For realization of the outlined multi-focus situation, the design approach used here employs the scalar Huygens-Fresnel principle in the context of discrete point holography^25,26^: Via the superposition of spherical elementary waves originating from the aperture plane that contains the phase mask, formation of individual focal spots in one or more desired image planes is obtained.

### 3D multi-focus holograms

In order to calculate the phase distribution of the mask $\phi \left( {x,y,0} \right)$ within the aperture plane located at $\left( {x,y,z = 0} \right)$, the multi-focus distribution is defined via considering $N$ discrete foci located at coordinates $\left( {x_{j} ,y_{j} ,f_{j} } \right)$ in the image planes $z = f_{j}$. The phase distribution is mathematically represented as the phase of the complex electric field distribution in the aperture plane, originating from the superposition of $N$ elementary waves, each of which originates from one focal spot^25,26^:

1 $$\phi \left( {x,y,0} \right) = \arg \sum\nolimits_{j}^{N} { A_{j} \exp \left( { - ik\left[ {\sqrt {\left( {x - x_{j} } \right)^{2} + \left( {y - y_{j} } \right)^{2} + f_{j}^{2} } - f_{j} } \right]} \right) \in \left[ {0,2\pi } \right)} .$$

here $k = 2\pi n/\lambda_{0}$ is the wavenumber in a medium with refractive index $n$ ($\lambda_{0}$: vacuum wavelength). In Eq. (1), each summand of the series represents an individual spherical wavelet with its origin located at coordinates $\left( {x_{j} ,y_{j} ,f_{j} } \right)$, having a corresponding amplitude $A_{j}$. The latter can be chosen freely and therefore allows investigating specific phase configurations that cannot be straightforwardly realized via iterative approaches. Note that Eq. (1) uses a scalar representation, however, extending it to a full vectorial description of the complex electric field even allows to account for its polarization^37,38^. Nevertheless, for sake of simplicity, here we decided to only treat the scalar propagation of waves. As a novelty over already existing approaches^25,26^, in the present study, we focus on two specific scenarios, where the phases of adjacent foci have different relationships: (1) in-phase configuration ($\uparrow \uparrow$): $A_{j} = 1$ for all $j$, and (2) opposite-phase configuration ($\uparrow \downarrow$): $A_{j} = \pm 1$, corresponding to alternating phases between adjacent foci. The printed polymer structure is a circular disk of 60 μm diameter with a height profile defined by $h\left( {x,y} \right) = \phi \left( {x,y} \right)\lambda_{0} /\left( {2\pi \left( {n - 1} \right)} \right)$. Here φ(*x,y*) is the phase profile according to Eq. (1) and *n* the refractive index of the polymer at the operation wavelength λ_0_.

### Influence of phase

The minimum resolvable center-to-center distance $d_{min,j}$ between two foci $j_{1}$ and $j_{2}$ in the image plane $f_{j}$ is given via the Abbe resolution limit^39^ $d_{min,j} \approx \frac{{1.22\lambda_{0} }}{{2NA_{j} }}$, where the numerical aperture of the system is $NA_{j} = n\sin \left[\arctan \left( {\frac{D}{{2f_{j} }}} \right) \right] = \frac{nD}{{\sqrt {D^{2} + 4f_{j}^{2} } }}$ with aperture diameter *D*.

**Figure 2:**
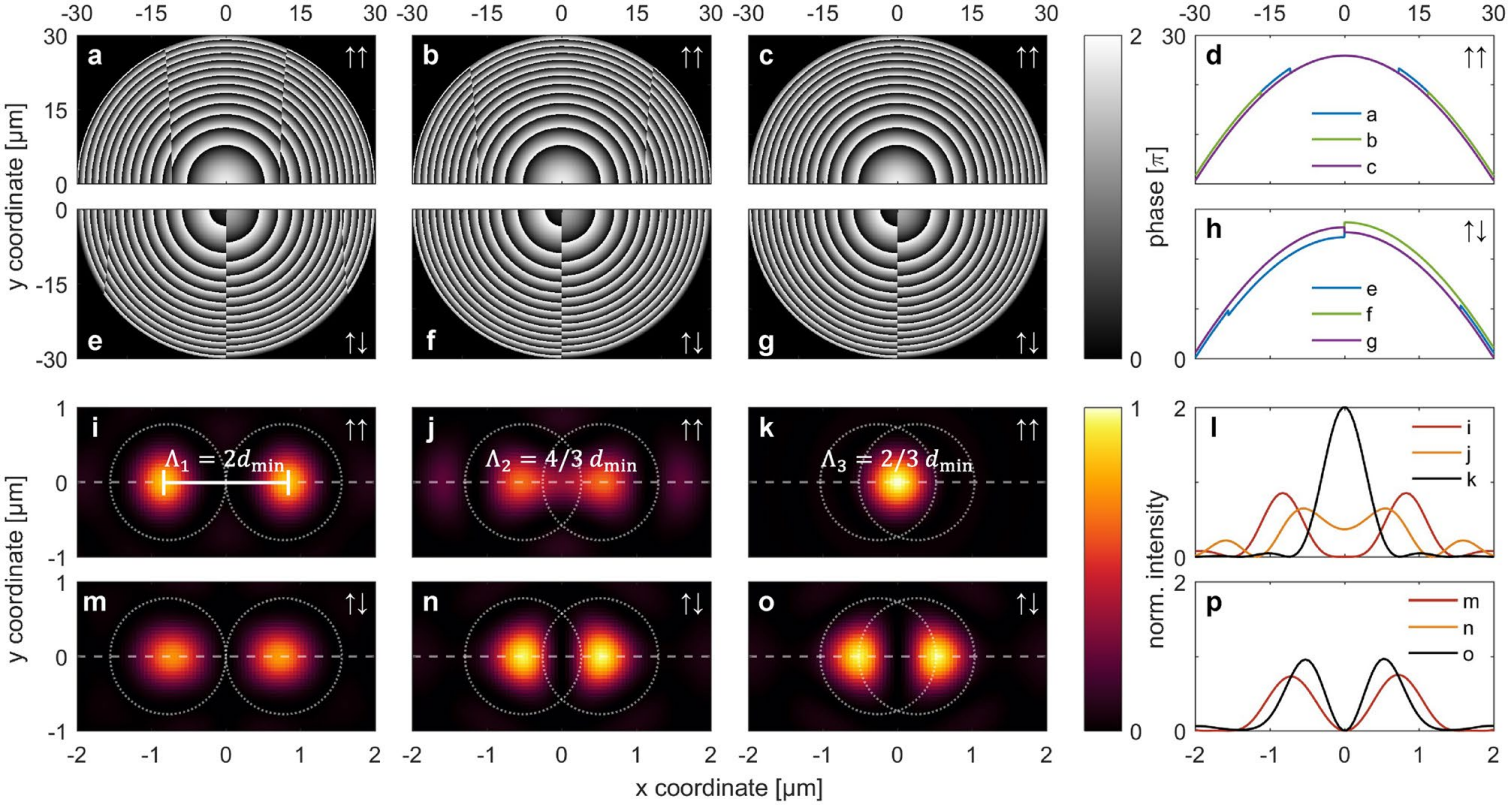
Comparison of in-phase (↑↑) and opposite-phase (↑↓) configuration for the situation of two foci (*N* = 2) for different inter-focal distances *Λ*. (**a**)–(**c**) Phase holograms in the aperture plane (*z* = 0) for the ↑↑ case: (**a**) *Λ*_1_ = 2 *d*_*min*_, (**b**) *Λ*_2_ = 4/3 *d*_*min*_, (**c**) *Λ*_3_ = 2/3 *d*_*min*_*,* together with their unwrapped phase profiles (having the 2π modulus removed) along *y* = 0 shown in (**d**). (**e**)–(**h**) Respective phase distributions for the ↑↓ scenario. (**i**)–(**k**) Intensity distributions in the focal plane (*z* = *f*) obtained from the holograms shown in (**a**)–(**c**) (↑↑ case). (l) Corresponding profiles along *y* = 0 direction (horizontal dashed lines in (**i**)–(**k**)). (**m**)–(**p**) Corresponding distributions for the ↑↓ case (scenarios (**e**)–(**h**)). Note that the yellow curve in (p) is identical to and lies underneath the black curve. The maximum intensity (*I*_0_) of all double focus distributions was used for normalization of the data in (**i**), (**j**), and (**l**)–(**p**). For better visibility, the data in (**k**) were normalized by 2*I*_0_.

**Figure 3:**
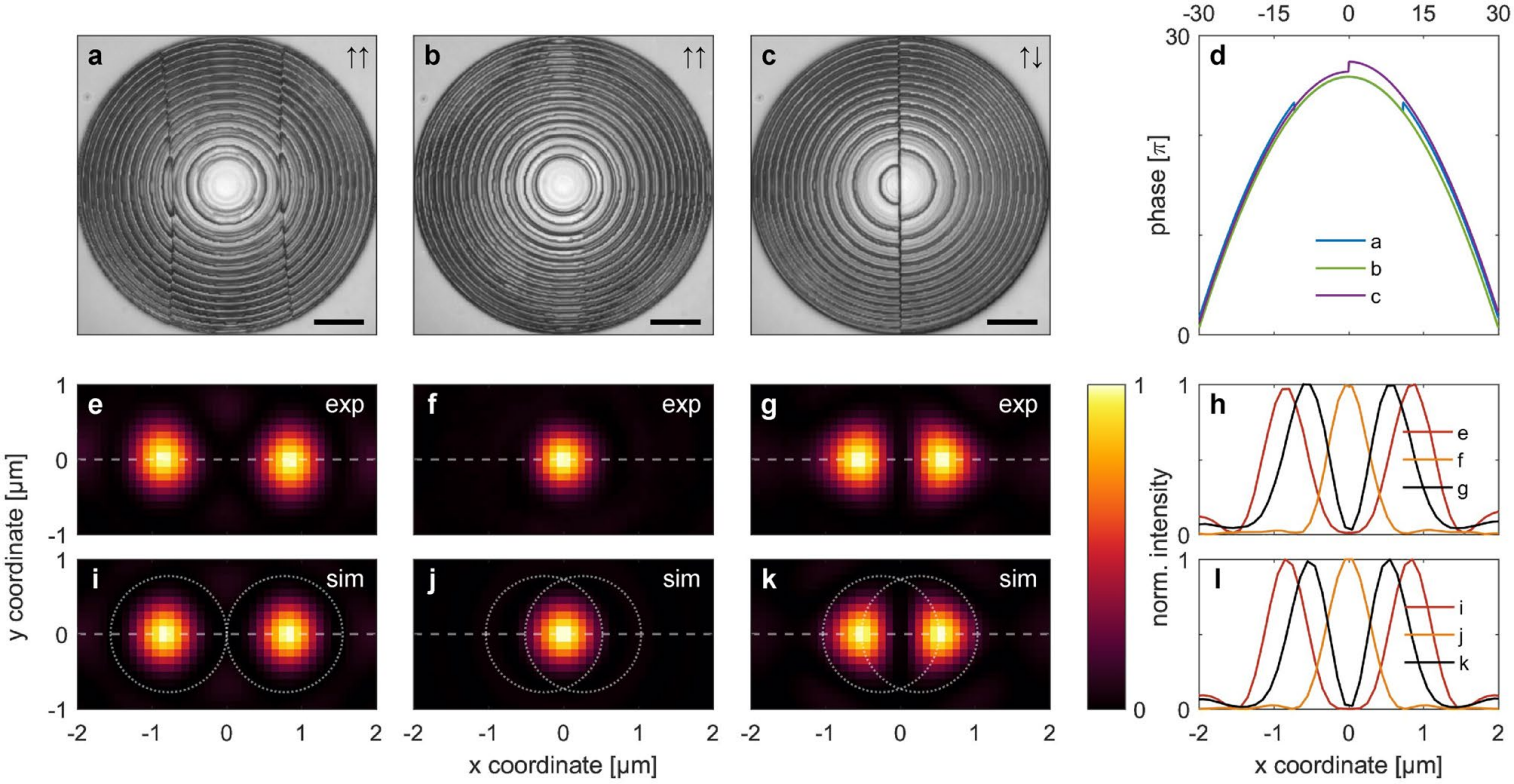
Nano-printed CGHs and measured dual-focus intensity distributions in the focal plane of three selected in—and opposite-phase configurations in comparison to simulations. (**a**)–(**c**) Experimentally realized 3D nano-printed holograms in the aperture plane: (**a**) $\uparrow \uparrow /\varLambda_{1}$, (**b**) $\uparrow \uparrow /\varLambda_{3}$, (**c**) $\uparrow \downarrow /\varLambda_{3}$ (scale bar 10 µm). (**d**) Corresponding unwrapped phase profiles of the distributions shown in (**a**)–(**c**) along $y = 0$. (**e**)–(**g**) Measured intensity in the focal plane from the holograms shown in (**a**)–(**c**) with the intensity profiles along $y = 0$ (horizontal dashed lines) shown in (**h**). (**i**)–(**l**) Corresponding simulated patterns for the scenarios in (**e**)–(**h**).

As a first case, we concentrate on the situation of two foci ($$N = 2$$) within a single image plane located at $${\text{z}} = f$$: Based on the above-mentioned procedure, Fig. 2 shows a comparison of the simulated phase distributions $$\phi \left( {x,y,0} \right)$$ in the aperture plane (at $$z = 0$$) for the in—and opposite-phase scenarios ($$\uparrow \uparrow$$ and $$\uparrow \downarrow$$) for three selected inter-focal distances *Λ* (center-to-center, $$\varLambda_{1} = 2d_{\min }$$, $$\varLambda_{2} = 4/3 d_{min}$$, $$\varLambda_{3} = 2/3 d_{min}$$) together with the resulting intensity distributions in the image plane (an overview of all treated scenarios in this work can be found in Supplementary Table S1 in the Supplementary Information). Therefore, the considered simulation parameters have been fixed as $$NA = 0.5$$, $$D = 60 {\mu m}$$ (corresponding to $$f = 52 {\mu m}$$), $$\lambda_{0} = 637 {\text{nm}}$$, and $$n = 1$$ (air), leading to a minimum resolvable distance of two adjacent focal spots (Abbe limit) of $$d_{min} = 777 {\text{nm}}$$. Note that the values of the inter-focal distance are chosen to represent the different cases: (1) $$\varLambda_{1} > > d_{min}$$, corresponding to largely separated and thus well resolved focal spots (Fig. 2a,e,i,m), (2) $$\varLambda_{2} \underset{\raise0.3em\hbox{$\smash{\scriptscriptstyle\thicksim}$}}{ > } d_{min}$$, representing the case of a focal separation slightly above the resolution limit (Fig. 2b,f,j,n), and (3) $$\varLambda_{3} \underset{\raise0.3em\hbox{$\smash{\scriptscriptstyle\thicksim}$}}{ < } d_{\min }$$, referring to the situation just below the critical resolution $$d_{min}$$ (Fig. 2c,g,k,o).

The simulation results reveal an interesting evolution: As expected, above the resolution limit (*Λ*_1_, Fig. 2i,m), two clearly separated focal spots are observed for both phase scenarios. In case the center-to-center distance approaches the critical limit $d_{min}$ (*Λ*_2_), the two foci start to fuse for the in-phase ($\uparrow \uparrow$) situation (Fig. 2j), while in the opposite-phase case ($\uparrow \downarrow$) they remain well resolved (Fig. 2n). For inter-focal spacing below $d_{min}$ (*Λ*_3_), the two spots completely fuse into one single spot of twice the intensity than for the in-phase ($\uparrow \uparrow$) case (Fig. 2k). In the opposite-phase scenario ($\uparrow \downarrow$), remarkably, the foci remain well resolved even though being designed as below the resolution limit ($\varLambda < d_{min}$, Fig. 2o). Note that when comparing Fig. 2f–g, the non-visible difference between the phase distributions of the opposite-phase cases ($\uparrow \downarrow$) for $\varLambda_{2}$ and $\varLambda_{3}$ suggest that the corresponding intensity distributions in the focal plane (Fig. 2n–o) are also identical. In the supplementary material available we also show the related intensity distributions in the *xz*-plane (Fig. S2). Here we would like to mention that the phases of the resulting interference patterns are given by Eq. (1) and consist of circular patterns arising from the interference of each spherical wavelet with a plane wave, while the hyperbolic curves correspond to the interference of both spherical waves. The latter leads to the phase discontinuities shown by the kinks in Fig. 2d,h. The simulated intensity distributions in the focal plane were also used to calculate the diffraction efficiency. Here we use the efficiency criterion given by Arbabi^40^, which is based on a circular area with a radius of 3 times the FWHM around the focus. The efficiency is defined as the power passing through this area normalized by the initial power passing the metasurface. The FHWM values for the different cases shown in Fig. 2 are 0.63 ± 0.06 μm. The related efficiencies are in the range of η = 83–92%.

## Experimental results

The experimental realization of the CGHs relies on two-photon lithography-based femtosecond direct laser writing (fs-DWL or 3D optical nano-printing) with a commercial system (Photonic Professional GT2, Nanoscribe GmbH). Besides advantages such as speed, reliability and cost efficiency, this implementation approach enables the direct realization of nanostructures on optical fibers while avoiding additional post-processing or clean-room-involving steps such as polishing, metal mask deposition etc. To evaluate the presented complex phase included Huygens-Fresnel principle-based design concept, various multi-focus holograms (Fig. 2) were 3D nano-printed on fused silica substrates via a 1.4NA dip-in objective using IP-Dip photoresin (Nanoscribe GmbH). For the demonstration of the remote beam shaping capabilities, a complex field distribution in two image planes was realized on a standard single-mode fiber in a second step via the implementation of a sophisticated CGH. Note that silica was chosen as the substrate to mimic the situation of the glass fiber surface. All holograms exhibit a circular shape with a diameter of $D = 60 \upmu m$, which is well compatible with the outer diameters of commonly used optical fibers. The operating wavelength was chosen to be $\lambda_{0} = 637 nm$ and air was assumed as the surrounding medium. Printing time per sample was approximately 30 min. In order to compensate for a possible tilt of the substrate and as well to increase adhesion (for details of the printing and the chemical development see Reference^33^), a 3 µm thick base layer was printed below the actual CGH, which only results in a constant phase offset being practically irrelevant. Note that here, no special adhesion promoter was used, and the total thickness of the hologram alone is approximately 1.2 µm, resulting from the refractive index contrast of the IP-Dip resin, modulated to a minimum individual height of each element via 256 different bitmap gray values with a spatial resolution of 50 nm.

According to the manufacturer the size of the printed ellipsoid shaped voxels is 500 nm in lateral and 1500 nm in vertical direction. The positioning step size can be selected much smaller which allows us to print the saw-tooth-like radial profile of our lenses having a minimum radial pitch of approximately 1 μm in the outer region with sufficient precision. Features with heights < 1500 nm can be printed by moving the laser focus partially into the substrate. The structures shown here were printed with 24% of the full laser power and a scan speed of 3600 μm/s.

The optical characterization was performed via illuminating the samples with a collimated laser beam (Thorlabs LP637-SF70) of diameter much larger than the holograms and imaging the intensity distribution in the focal plane via a home-built microscope (Olympus MPLFLN50x + Thorlabs AC254-200-A-ML) onto a CMOS camera (Thorlabs DCC1545M).

### Linear dual-focus

In order to experimentally verify our simulation results and particularly to show the different behavior of the in—and opposite-phase CGHs, the designs shown in Fig. 2 were nano-printed ($NA = 0.5$), and the intensity distribution in the focal plane was optically characterized. To avoid redundancy, three designs have been selected and are compared in Fig. 3 to the corresponding simulated distributions (Fig. 3a,e,i: $\uparrow \uparrow$/$\varLambda_{1}$; Fig. 3b,f,j: $\uparrow \uparrow$/$\varLambda_{3}$; Fig. 3c,g,k: $\uparrow \downarrow$/$\varLambda_{3}$).

Overall, the measurements reproduce the simulated results excellently: Above the critical distance, both foci are well resolved and behave according to the simulations when $\varLambda$ decreases. The designs proposed in Fig. 2 are thus fully reproduced, which underlines the relevance of 3D nano-printing for the realization of CGHs.

### Circular multi-focus arrangement

**Figure 4:**
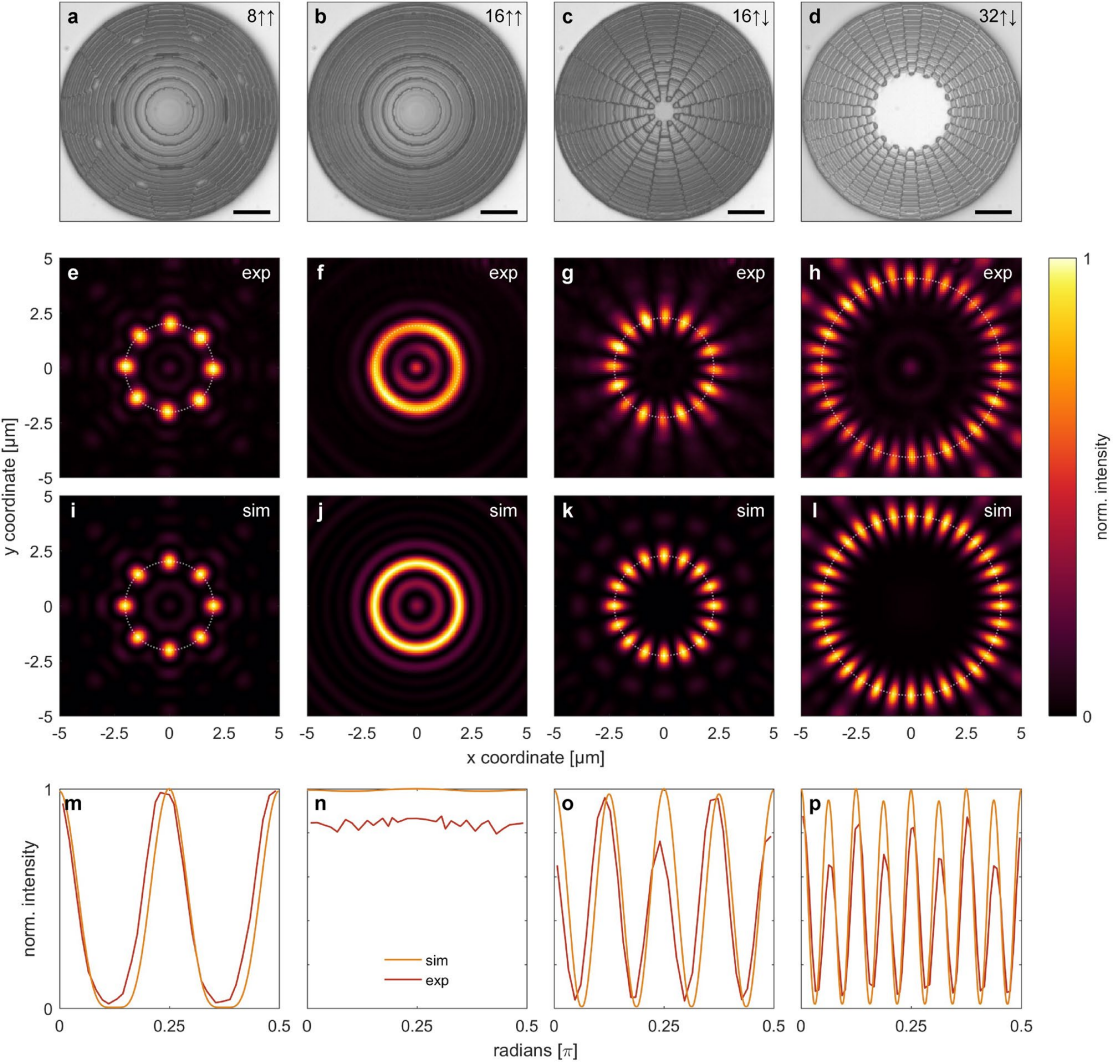
(**a**)–(**d**) Selected examples of implemented CGHs, (**e**)–(**h**) measured and (**i**)–(**l**) simulated intensity distributions in the focal plane for the situation where a discrete number of foci are located on an annulus of circumference $C = 16 d_{min}$. (**m**)–(**p**) show the corresponding comparisons of the azimuthal intensity distributions along the white dotted circles. Each column refers to a different configuration (from left to right): (**a**), (**e**), (**i**), (**m**) $N = 8$, $\varLambda_{1} = 2 d_{min}$, $\uparrow \uparrow$; (**b**), (**f**), (**j**), (**n**) $N = 16$, $\varLambda_{2} = 4/3 d_{min}$, $\uparrow \uparrow$; (**c**), (**g**), (**k**), (**o**) $N = 16$, $\varLambda_{2} = 4/3 d_{min}$, $\uparrow \downarrow$; (**d**), (**h**), (**l**), (**p**) $N = 32$, $\varLambda_{3} = 2/3 d_{min}$, $\uparrow \downarrow$. The scale bars in the top row refer to 10 µm.

As a next step, we extend the previous study to a chain of foci arranged on an annulus of circumference $C$, which is correlated to the inter-focal distance and number of foci by $C = N \cdot \varLambda$. This definition imposes all inter-focal distances to be identical and allows relating this investigation to the previous dual-focus study by choosing the already introduced values of $\varLambda$ (Fig. 2). In this context, it is particularly interesting to study the behavior of the system when more foci are added to the chain for the same value of $C$. In detail, the number $N$ of foci is increased while the circumference remains constant, so that the inter-focal distance becomes smaller than the resolution limit ($\varLambda < d_{min}$). In light of this, the measured and simulated intensity distributions of four different multi-focus scenarios ($NA = 0.5$, $C = 16 d_{min}$) are compared in Fig. 4: (1) $N = 8$, $\varLambda_{1} = 2 d_{min}$, $\uparrow \uparrow$ (Fig. 4a,e,i,m), (2) $N = 16$, $\varLambda_{2} = 4/3 d_{min}$, $\uparrow \uparrow$ (Fig. 4b,f,j,n), (3) $N = 16$, $\varLambda_{2} = 4/3 d_{min}$, $\uparrow \downarrow$ (Fig. 4c,g,k,o), and (4) $N = 32$, $\varLambda_{3} = 2/3 d_{min}$, $\uparrow \downarrow$ (Fig. 4d,h,l,p). An overview of the considered scenarios can be found in Supplementary Table S1, and the corresponding simulated phase distributions can be found in Supplementary Fig. S1 within the Supplementary Information. Before going into detail, it is important to note that all simulations are fully reproduced by the experiments (middle and bottom rows in Fig. 4), again emphasizing the excellent implementation capabilities of the 3D nano-printing approach.

In case the inter-focal distance is above the resolution limit for the in-phase configuration ($\uparrow \uparrow$, $\varLambda_{1} = 2d_{min}$, Fig. 4e,i), the individual foci on the circumference are well resolved in agreement with the observation of the dual-focus study (Fig. 2i) and match the simulation (Fig. 4m). In accordance with the distribution shown in Figs. 2j, 4f,j reveal that the individual focal spots cannot be resolved anymore and mutually merge when the inter-focal distance approaches the resolution limit ($\varLambda_{2} = 4/3d_{min}$) for the in-phase ($\uparrow \uparrow$) configuration. Note that this behavior can already be anticipated from the appearance of the corresponding hologram (Fig. 4b) which reveals no azimuthal dependence. The measured profile intensity has a lower amplitude than its corresponding simulated counterpart, which we attribute to a tilted substrate during the measurement and thus different focal plane positions of the individual foci along axial direction. The behavior of the opposite-phase configuration ($\uparrow \downarrow$), conversely, shows well-separated foci on the circumference, again in accordance with the results of the dual-focus study (Fig. 2n). Interestingly, a further increase in the number $N$ of foci imposes the diameter of the ring to transversely extend (Fig. 4h,l). This effect is remarkable, as the design considerations assume the circumference of the annulus to remain unchanged ($C = 16d_{min}$), presumably being associated with the tendency of the optical system to preserve the critical distance between individual foci. This particular configuration leads to unexpected effects when successively increasing the number of foci and will be investigated in more detail in a future study. This effect is described in more detail in Supplementary Figure S3. Nevertheless, both configurations (Fig. 4o–p) are in good agreement with the simulations regarding periodicity and thus resolution of the individual focal spots. In particular, the alternating sequence of maximum intensity along the white circle is also found in both the simulation and the experiment, while the slight deviations in the relative intensity distributions are again due to tilting of the substrates.

### Fiber-based 3D multi-focus holography

**Figure 5:**
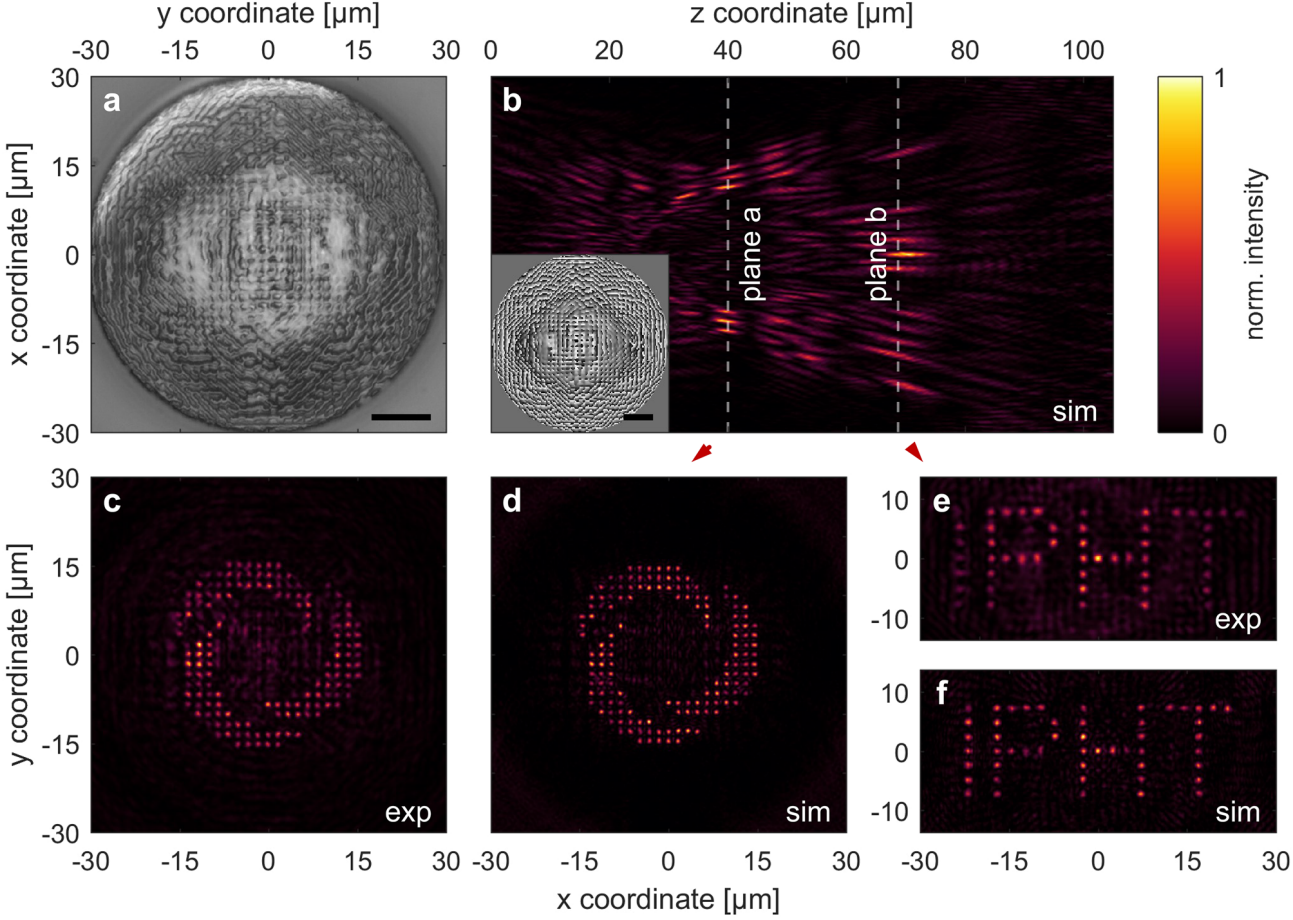
Implemented CGH (**a**) and measured/simulated intensity distributions (**b**)–(**f**) of a fiber-interfaced 3D multi-focus hologram, creating 192 foci in two image planes behind a single-mode fiber. (**a**) Experimentally realized 3D nano-printed CGH on the facet of an SMF containing a core-less beam expansion section (scale bar 10 µm). (**b**) Simulated intensity distribution along the beam propagation axis (*xz*-plane). The inset shows the simulated CGH in the aperture plane at $z = 0$ (scale bar 10 µm). The image (*xy*-) planes are located at $f_{a} = 40 \mu m$ and $f_{b} = 70 \mu m$ behind the facet. (**c**, **d**) Measured and simulated intensity distribution in the first focal plane, showing the logo of the authors’ institution ($N_{a} = 142$, $NA_{a} = 0.6$). (**e**, **f**) Measured and simulated patterns in the second plane, showing the institute’s lettering ($N_{b} = 50$, $NA_{b} = 0.4$).

In order to demonstrate a pathway how the discussed approach can be used for remote holography, a complex phase CGH that generates more than a hundred spots in two image planes was interfaced with a commercially available single-mode fiber. It should be noted that the single-mode condition imposes limits on the diameter of the fiber core and thus the transverse extent of the fiber mode. In the present study, we chose a Thorlabs 630HP single-mode fiber, having a mode diameter of about 4 µm. In order to illuminate the entire aperture of the CGH ($D = 60 \mu m$), a section of a core-less fiber (length $L = 590 \mu m$) was spliced to the end of the SMF to sufficiently expand the emitted Gaussian beam that originates from the fundamental HE_11_ fiber mode (details of the procedure and the explicit implementation of the fiber wavefront can be found in Reference^16,33^). The CGH was designed such that the final 3D intensity distribution contains a total of $N = 192$ individual foci within two image planes behind the fiber facet ($z = 0$, Fig. 5b, d, f). The foci arrangement was chosen to resemble the logo of the authors’ institution—the Leibniz Institute of Photonic Technology (IPHT)—in the first plane (focal distance $f_{a} = 40 \upmu \text{m}$, Fig. 5c–d), while the corresponding lettering is shown in the second plane (focal distance $f_{b} = 70 {\mu m}$, Fig. 5e–f). The logo is composed of $N_{a} = 142$ individual foci with an inter-focal distance of $\varLambda_{a} = 2.5 d_{min,a} = 1.6 \mu m$ ($NA_{a} = 0.6$), while the lettering in the second plane contains $N_{b} = 50$ individual spots pitched at $\varLambda_{b} = 2.5 d_{min,b} = 2.4 {\mu m}$ ($NA_{b} = 0.4$).

The measurements (Fig. 5c,e) agree well with the corresponding simulations (Fig. 5d,f), thus demonstrating the full 3D capability of the presented multi-focus holography approach to distribute a multitude of foci in a defined manner over multiple image planes in case the inter-focal distance is larger than the resolution limit. Note that this also applies to the longitudinal spacing between individual foci along the optical axis, thus imposing limits to the axial spacing of adjacent planes. Nevertheless, the here presented concept allows for straightforward adaptation to a multitude of image planes while bearing in mind the aforementioned limits as well as the possibility for opposite-phase implementation. In principle, the deviations of the experimental results from the simulations are minor and can be explained as follows: (1) a general degradation of quality is found from the center towards the edges of the intensity pattern, presumably due the only scalar description of Eq. (1) in contrast to a full vectorial approach that accounts for oblique incidence at larger angles (higher NAs), and (2) a smearing of the theoretically staircase-shaped CGH and its surface roughness in the order of the printing resolution, which thus leads to a loss of contrast and stray light. We would also like to point out that the CGH here (Fig. 5a) looks rather asymmetrical in contrast to the previous counterparts. Thus, the good agreement of the simulation with the experiments again underlines the capabilities of the 3D nano-printing process for correct implementation of CGHs even in case of highly irregular surfaces.

## Discussion

### Further potential for remote beam shaping

In addition to published work^25,26,33,36^, our study clearly shows the potential of phase engineered and fiber-interfaced complex field CGHs, while we would like to highlight the use of a single-mode fiber. This type of fiber offers decisive advantages such as bending insensitivity, defined optical modes, and light transportation over extremely long distances, thus being ideal with regard to remote digital holography. An extension of the functionality portfolio can be achieved via the use of microstructured fibers such as multicore fibers. For instance, the tailored manipulation of the focal properties can be anticipated via changing the light that is coupled into different cores.

We would also like to emphasize the 3D nano-printing process used here, which, in contrast to processes such as modified e-beam lithography^27,41^ or self-assembly^29^, enables time-effective, tailored, and direct realization of selected nanostructures on fiber end-faces.

### Comparison to iterative approaches

Compared to iterative methods, the approach for designing CGHs presented here offers the advantage of a simple, robust, fast, and reliable calculation of a single and thus optimal phase distribution. In addition, from our perspective, the outstanding feature of our approach is the possibility to explicitly determine the phase of individual foci, and thus of the overall phase distribution, in addition to the intensity. This feature enables control over the phase and thus polarization of individual focal spots in an array of foci via extending Eq. (1) to a full vectorial description, which cannot be achieved via commonly used iterative phase optimization methods, as these only address the intensity in almost all cases.

We would like to clearly note here that the presented approach generates a special kind of complex phase-engineered CGH, being unique and different from those commonly employed. This suggests that our approach will not replace successful iterative methods for hologram optimization (e.g., Gerchberg-Saxton algorithm^17,18^), since a realization of a more continuous intensity distribution is not compatible with the presented approach.

The kind of CGHs presented here, is based on the generation of a discrete number of spherical wavelets that propagate towards the related foci. As long as the foci are sufficiently distant from each other, as well as their number is sufficiently small, the field of the other partial waves has only a negligible contribution to the focus of interest. Note that this condition is no longer fulfilled if a continuous intensity distribution is required. Thus, in this context, the presented method should be seen as a supplement to established methods and can be applied in situations where additional phase control is required.

### Potential multi-focus applications

Due to the unique properties of the presented concept, we believe it to be relevant for a variety of applications where binary—and complex phase-type arrangements of light fields are required. For example, the use of linear foci plays a key role in integrated microfluidic devices, for instance, where scattered, fluorescent, or Raman-shifted light from passing micro—and nano-objects (e.g., cells) can be effectively detected^42^. Applications of linear or more complex focal distributions in telecommunications for efficient simultaneous in-coupling into multicore fibers can also be anticipated^10^. Furthermore, arranging multiple foci, each having a high *NA* (> 0.8), allows the realization of optical multi-site traps aiming at specific applications in fields such as quantum technology (e.g., trapping of single emitters in cryogenic environments^43^) or life sciences (e.g., parallel detection of nanoscale species^44^).

### Potential phase-sensitive excitation applications

Besides providing discrete foci, a key feature of the presented approach is the possibility to selectively adjust the relative phase of each individual spot. For example, the concept can be used to tailor light fields in order to selectively excite resonances with sophisticated mode and phase or polarization distributions in optical systems with high efficiency via including a vectorial description of Eq. (1). Examples of such resonances are guided higher-order modes in waveguides (e.g., LP-modes in solid glass^8^ and hollow-core fibers^9^ or plasmonic excitations on nanowires^45^), and localized resonances (e.g., localized plasmon modes^46^ and dielectric metasurfaces^47^).

### Potential applications using fibers

In all these applications, the use of devices that are interfaced with single-mode fibers suggests a significant improvement in the degree of integration and a decisively simplified handling, as demonstrated in applications such as nanoparticle tracking^48^, optical trapping^33^, and bio-sensing^49^ and imaging within optical coherence tomography endoscopy^50^. For instance, fibers allow transportation of light over remote distances almost loss-free and bend-insensitive to predefined locations, for example making mirror-based optics obsolete in scanning applications.

## Conclusion

The desired generation of tailored light field patterns with complex spatial distribution, including intensity as well as phase control, is essential within many areas of science and technology. In this context, we present here a concept that allows for the realization of multi-focus intensity patterns with additionally adjustable relative phases between individual foci via directly applying an extension to Huygens-Fresnel principle. We demonstrate, in simulations and experiments, control of the phase of individual foci in an array of focal spots that is three-dimensionally distributed. Contrary to iterative methods, our complex phase-engineered interference-based design principle enables sophisticated computer-generated holograms for realizing unconventional scenarios, such as opposite phases of neighboring foci. Using this, we demonstrate that two foci can still be separated, although their distance is designed to be below the resolution limit. Experimental verification was realized via implementing different CGHs on different substrates using 3D nano-printing, showing an excellent match between the design and the implemented structures. In addition to planar substrates, 3D holograms were created on the end-faces of modified single-mode optical fibers, generating intensity distributions consisting of about 200 individual foci distributed over two image planes, with an extension of the presented approach to more planes being straightforwardly possible via implementing a full vectorial description.

The presented approach offers numerous unique advantages, such as robust, stable, and straightforward 3D digital holography over remote distances, as well as precise control over intensity and phase of each focus in an array of three-dimensionally arranged spots. In particular, phase (and thus polarization) control is difficult to achieve using iterative optimization methods, suggesting enormous potential applications in fields such as quantum technology, life sciences, bioanalytics and telecommunications. In particular, the presented phase control enables the excitation of higher-order optical resonances, being highly relevant in various fields such as nanophotonics, fiber optics and waveguide technology.

## Supplementary Information

Supplementary Information 1.

Supplementary Information 2.

Supplementary Information 3.

Supplementary Information 4.

## Data Availability

All data generated or analyzed during this study are included in this published article (and its Supplementary Information file).
