# Supplementary material for: Fiber-based 3D nano-printed holography with individually phase-engineered remote points
Source: Sci Rep. 2022 Dec 3;12:20920. doi: 10.1038/s41598-022-25380-2 (PMC9719565; doi:10.1038/s41598-022-25380-2)
Supplement: Supplementary file 4 — Supplementary Information 4. [file 41598_2022_25380_MOESM4_ESM.pdf]

Supporting Information

Fiber-based 3D nano-printed holography with individually phase-engineered remote points

Malte Plidschun[1], Matthias Zeisberger[1], Jisoo Kim[1,2], Torsten Wieduwilt[1], and Markus A. Schmidt[1,2,3,*]

[1]Leibniz Institute of Photonic Technology, Albert-Einstein-Str. 9, 07745 Jena, Germany
[2]Abbe Center of Photonics and Faculty of Physics, Friedrich-Schiller-University Jena, Max-Wien-Platz 1, 07743 Jena, Germany
[3]Otto Schott Institute of Materials Research (OSIM), Friedrich-Schiller-University Jena, Fraunhoferstr. 6, 07743 Jena, Germany
*markus.schmidt@leibniz-ipht.de

Overview of implemented holograms

Table S1. Considered scenarios of discrete 3D multi-focus holograms with different inter-focal distances Λ and in- and opposite-phase configurations.

name of scenario	number of foci	inter-focal distance [d_{min}]	phase symmetry	shown in Figure
linear dual-focus	2	2 (0.5 NA)	↑↑	2a,i, 3a,e,i
			↑↓	2e,m
		4/3 (0.5 NA)	↑↑	2b,j
			↑↓	2f,n
		2/3 (0.5 NA)	↑↑	2c,k, 3b,f,j
			↑↓	2g,o, 3c,g,k
circular multi-focus	8	2 (0.5 NA)	↑↑	4a,e,i
	16	4/3 (0.5 NA)	↑↑	4b,f,j
			↑↓	4c,g,k
	32	2/3 (0.5 NA)	↑↓	4d,h,l

3D fiber multi-focus	192	2.5 (mixed NA)	↑↑	5
	142	(0.6 NA)	↑↑	5c,d
	50	(0.4 NA)	↑↑	5e,f

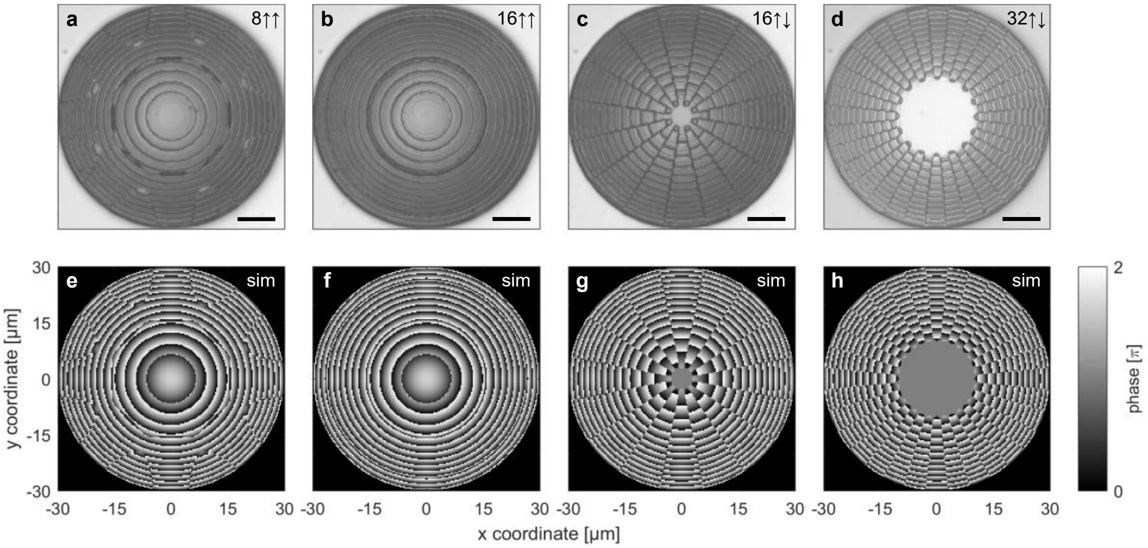

Figure S1. (**a**)–(**d**) Selected examples of implemented holograms and (**e**)–(**h**) simulated phase distributions in the aperture plane for the situation where a discrete number of foci are located on an annulus of circumference $C = 16\ d_{\mathrm{min}}$. Each column refers to a different configuration (from left to right): (**a**), (**e**) $N = 8$, $\Lambda = 2\ d_{\mathrm{min}}$, ↑↑; (**b**), (**f**) $N = 16$, $\Lambda = 4/3\ d_{\mathrm{min}}$, ↑↑; (**c**), (**g**) $N = 16$, $\Lambda = 4/3\ d_{\mathrm{min}}$ ↑↓; (**d**), (**h**) $N = 32$, $\Lambda = 2/3\ d_{\mathrm{min}}$, ↑↓. The scale bars in the top row refer to 10 µm.

Focus fields in the xz-plane

As a complement to the intensity distributions of the dual focus arrangements in the *xy*-plane which are shown in Fig. 2 (i-k, m-o) the related distributions in the *xz*-plane are shown in Fig. S2.

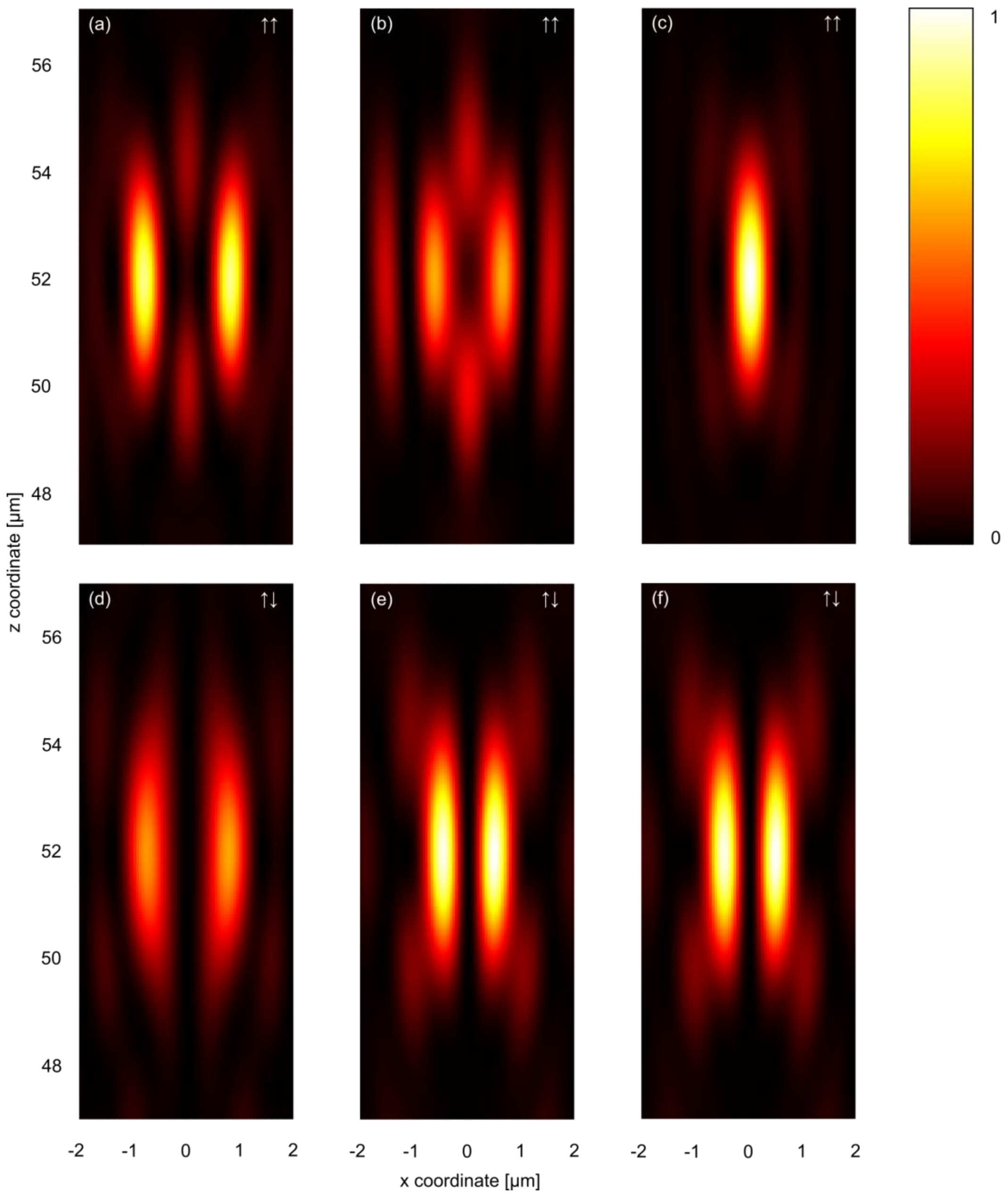

Figure S2. Comparison of simulated intensity distributions in the xz-plane near the axial focal distance (f=52 µm) for the situation of two foci (N=2). The designed lateral distances between the two foci are Λ =2 d_{min} (a,d), Λ= 4/3 d_{min} (b,e), and Λ =2/3 d_{min} (c, f). The upper row (a-c) shows the result for the foci being in phase (↑↑), the lower row for the out off phase (↑↓) case. Intensities are normalized in the same way as in Fig. 2 of the main text.

Focal shift to larger radii

All configurations presented in Fig. 4 of the main text were designed using Eq. (1) to have the foci located on a circle of radius $r = 2$ µm in the focal plane. The simulation and the measurements of the associated intensity distributions correspond well to this design parameter for $N = 8$ and $N = 16$ foci. For $N = 32$ foci with alternating phases, however, the foci are shifted to much larger radii of $r = 4$ µm. To understand this effect in more detail, we performed simulations for several numbers of foci between $N = 16$ and $N = 32$ in the in- and opposite-phase configuration. All other parameters are identical to those previously used. The results are presented in Fig. 7.

For the in-phase scenario, the data clearly shows that the foci are merging but are still located approximately at the designed radius of $r = 2$ µm. In the opposite-phase configuration, however, the foci are gradually shifted to larger radii with increasing N. Figure 7u shows the radial positions for the different cases, and Figure 7v shows the inter-focal distance of adjacent foci in units of the resolution limit $d_{\min} = 777$ nm.

It is remarkable to note that for the opposite-phase configurations, the inter-focal distance always corresponds to approximately $d_{\min}$. Our interpretation of this effect is as follows: For $N > 16$, the inter-focal distance d of adjacent foci at radius $r = 2$ µm is $d < d_{\min}$. In the in-phase configuration, this leads to a merging of the foci into one circular focus. However, in the opposite-phase scenario, such a small distance of foci with opposite phases results in destructive interference. This leads to a shift of the foci to larger radii where they are still well separated ($d \cong d_{\min}$).

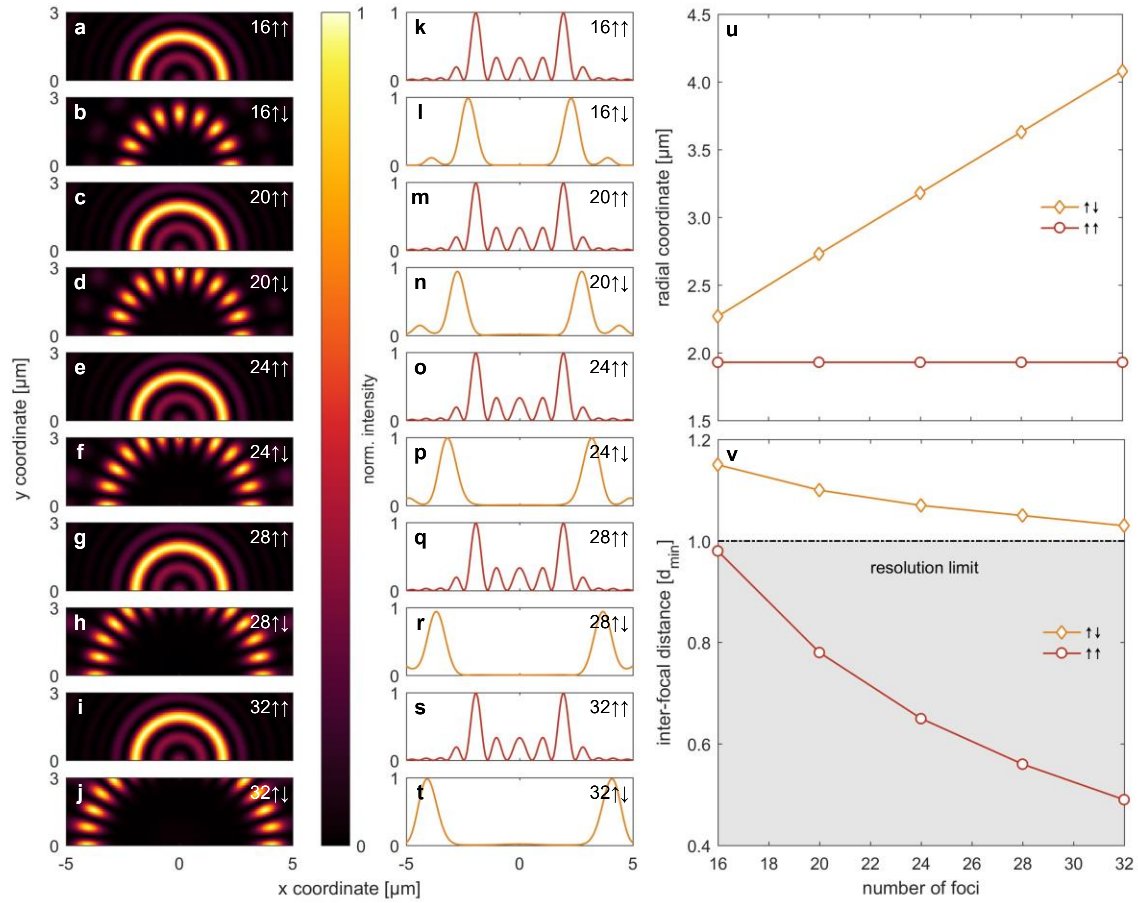

Figure S3. (**a**)–(**j**) Simulated intensity distribution within a cut-out of the xy-plane at focal distance ($z = f$) for several numbers $N = 20\ldots32$ of foci in the in- and opposite-phase configuration and (**k**)–(**t**) intensity along a line at $y = 0$. (**u**) Radial positions of the foci and (**v**) inter-focal distances of adjacent foci in units of the resolution limit $d_{\min} = 777$ nm for the different scenarios.
